# Machine learning for precision medicine: promoting value considerations through perspective-taking hypothetical group design exercises

**DOI:** 10.1007/s43681-025-00973-5

**Published:** 2026-02-01

**Authors:** Tehmi E. den Braven, Ariadne A. Nichol, Matthew D. Kearney, Mildred K. Cho, Pamela L. Sankar

**Affiliations:** 1 Center for Biomedical Ethics, Stanford University School of Medicine, Stanford, USA; 2 Department of Family Medicine and Community Health, Perelman School of Medicine, University of Pennsylvania , Philadelphia, USA; 3 Department of Medical Ethics and Health Policy, Perelman School of Medicine, University of Pennsylvania, Philadelphia, USA

**Keywords:** Machine learning, Precision medicine, Ethics, Responsibility, Developers

## Abstract

Public concerns over the social and ethical consequences of artificial intelligence (AI) are well established. Despite ongoing efforts to respond, these concerns remain largely unresolved by either regulation or codes of ethics. In response, scholars have advanced ideas about how to better ground ethical awareness in practice. A key element of this grounding is fostering awareness of how one’s actions can affect the welfare of others. We tested the effect of a group design exercise on whether and how AI developers considered the impact of their work on others, using perspective-taking as a “values lever”–a practice that prompts ethical reflection during the design process. We found that hypothetical scenarios set in three different contexts of AI research or building a tool for clinical use encouraged developers to take different perspectives. We specifically used an *imagine-self* framing to instruct AI developers to think about how they would feel or act in a certain situation. In progressing through the scenarios, developers’ design considerations shifted from methodological and data concerns to thinking about other interest holders, implementation, and social and ethical issues. In particular, a scenario that used the *imagine-self* framing appeared to lead to greater consideration of the patient perspective, self-awareness of this shift in perspective, and how it might and should affect their future practice. We conclude that a brief group exercise can increase awareness of the impact of design considerations on a broad range of interest holders, and inspire plans for action in future work.

**Supplementary Information:**

The online version contains supplementary material available at 10.1007/s43681-025-00973-5.

## Introduction

Public concerns over the social and ethical consequences of artificial intelligence (AI) are well established [1, 2]. Despite ongoing efforts to respond, however, these concerns are increasing and remain largely unresolved [1, 3–5]. For example, regulation has not kept pace with the novel challenges of evaluating AI, and recent policy mandates for “responsible AI” in the U.S. have been rolled back [6, 7]. Attempts to ensure ethical AI were otherwise focused on development of codes of ethics, until research to assess their effectiveness came up empty-handed [8–11]. Reflection on these findings led observers to conclude that the fault lay not in codes themselves but in inadequate knowledge among AI developers and others about how to apply them. Insight that ethical principles do not apply themselves [12] directed researchers to focus on what the field now considers the primary hindrance to effective AI ethics: a gap between ethical principles and their translation into practice in everyday design decisions [13–19].

Proposals to bridge this gap fall generally into two groups, distinguished by how they conceptualize the problem. Some scholars attribute the gap to specific deficiencies such as inaccessible or inadequate ethics-related information, and propose pragmatic responses like simplifying or systematizing ethics codes—for instance, by creating typologies that link ethical principles to “actionable solutions” [13, 14, 20, 21]. While simplifying and better organizing such resources is certainly useful, these information-based proposals often fall short in practical application. By focusing narrowly on information itself, they disregard the challenges AI developers (hereafter called machine learning developers, or MLDs) actually encounter when integrating ethics-informed choices into design, effectively treating information about a solution as the solution itself. Reliance on such strategies to bridge the principles to practice gap is likely to succeed only in stranding AI ethics on the practice side of the gap, asking, what now?

In contrast, scholars who see the problem as an expression of the field’s inadequate attention to ethics broadly favor plans that seek to better ground ethical awareness in practice and everyday work routines. They promote novel pedagogical methods or favor virtue ethics as the foundation of AI ethics [14, 22–25]. A critical challenge faced by all efforts to instantiate ethical norms, and underestimated in AI, is the need to foster a particular way of thinking. This way of thinking is the “awareness that something one might do… can affect the welfare of someone else” [26–28]. Research suggests that people working in AI and related fields might find this awareness particularly elusive [12, 29, 30].

Addressing such possible shortcomings is particularly urgent in health care AI, as health care fulfills a critical, broadly valued, societal function. Health care’s distinctive and prominent role is supported and reproduced through long accepted and extensively vetted ethical principles, which were developed to protect patients and promote population well-being. These principles and their underlying assumptions, which guide right decisions in health care, differ fundamentally from those in other domains. People trained to work in health care are trained in this knowledge. People working in computer science or in artificial intelligence generally are not. Addressing this deficit can contribute to successfully and ethically integrating AI into health care. Paradoxically, however, AI’s principles-to-practice literature has paid scant attention to the daily routines, attitudes, and practices of the developers who are tasked with integrating ethical principles into AI tools.

In the research reported here we describe a study that examines whether and how MLDs’ reasoning during hypothetical health care machine learning group design exercises expressed awareness of the existence and nature of the work’s potential consequences for others. We first provide background concerning our study design and theoretical framework, explaining the innovative method we use, developed to overcome challenges associated with values elicitation research [31, 32].

## Background

Recent scholarship argues that to effectively bridge the gap between principles and practice of AI development, efforts must shift from developing ethical algorithms to cultivating ethically-minded developers [33]. Ethical conduct requires awareness that one’s actions may have consequences for others. For AI developers, this means considering the rightness or wrongness of how design choices might impact the lives of people that a tool will affect. Sensitivity to one’s impact on others can be achieved through perspective-taking (PT), defined as learning to look at a situation from a viewpoint different from one’s customary viewpoint [34]. PT is understood to enhance empathy through a combination of cognitive and emotional processes that allow one to “feel another’s pain” while also distinguishing self from other [35]. PT exercises have been shown to influence individual moral attitudes and decision-making, with some studies demonstrating how framing can affect ethical reasoning in applied domains. For example, *veil-of-ignorance* adaptations have been used to study moral judgement in contexts such as autonomous vehicles and pandemic decision-making [36–39]. PT research sometimes distinguishes ways that an observer might be asked to imagine another’s world through *imagine-self* and *imagine-other* framing. *Imagine-other* instructs participants to think about how someone else would respond if they were the target or focus of an expressed attitude or action. *Imagine-self* instructs a participant to presume that the hypothetical attitude or action in question is being directed at themselves [40, 41]. For developers, this identification with the people whose lives are affected by a tool creates the potential for ethical reflection. Prompted by these findings, here we describe an innovative use of an *imagine-self* framing to explore whether and how developers’ reasoning during hypothetical design work for health care AI involves awareness of consequences to others. We also tested a simple intervention to enhance this awareness.

## Study design and methodology

The goal of this research was to examine whether introducing PT could encourage AI developers to bring ethical considerations to bear on their work. Two factors can hinder obtaining robust, authentic responses in this type of ethics-directed research. First, beliefs about what is and what is not ethical are infrequently discussed as such in everyday life and typically remain implicit. This makes their articulation on demand difficult, which can lead to superficial responses [42, 43]. Second, social desirability bias may encourage research participants to understate responses they perceive as undesirable and to emphasize responses they think will make them look good to investigators [44]. These factors argue against relying solely on direct queries into ethical beliefs or practices, and instead support collecting data under less structured circumstances designed to moderate a singular or explicit focus on ethics [45]. Research also has shown that actively engaging research participants in problem-solving encourages stronger efforts at reflection and sense-making than direct questioning alone [46].

Guided by these insights and our own experience in values elicitation research, we have developed a method that relies on group exercises (GEs) that actively engage participants in the type of activity we are examining. Instead of relying on direct questions about ethical beliefs or practices, we assign participants tasks that might introduce ethical concerns and observe ensuing interactions and then discuss choices they’ve made [31, 32]. Orientation and instructions for these GEs focus on the tasks, with minimal reference to ethics or values. During actual sessions we, as researchers, recede from center stage and leave participants to determine for themselves how to proceed. The objective is to encourage discussion more in line with how participants might handle similar problems in their real work and to reduce the likelihood of responses shaped by social desirability bias [45].

**Fig. 1:**
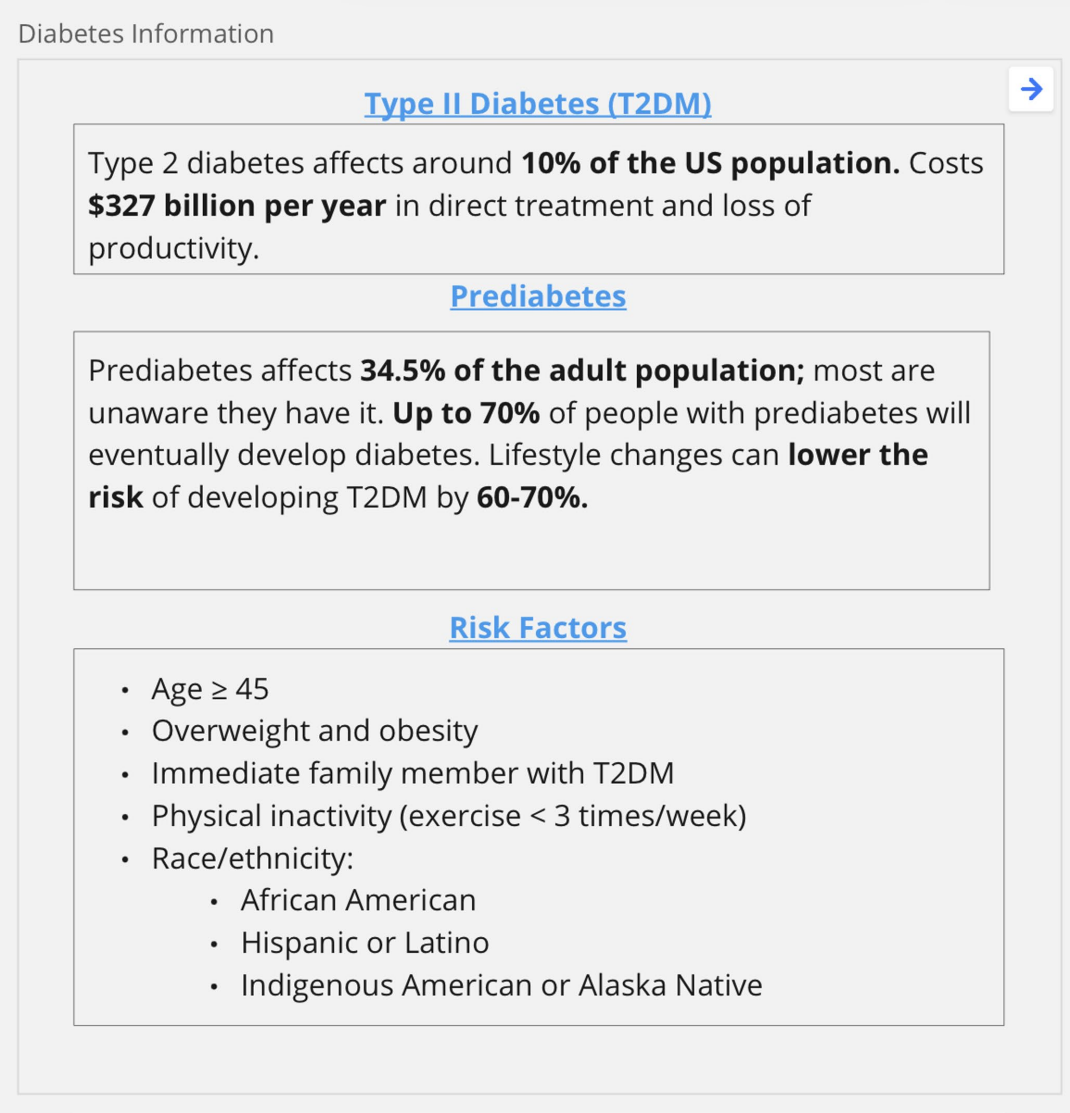
Diabetes background information provided during group exercise

For this project, we asked AI developers to work together on three machine learning design tasks related to the transition from prediabetes to diabetes in the United States. We chose this topic because diabetes is a widespread problem in the United States with which AI developers might be personally or professionally familiar. For orientation, we provided a table that listed select facts on the topic (see Fig. 1); importantly, however, completing the tasks required little to no medical knowledge. Features of the GE tasks intentionally corresponded to the inclusion criteria from our eligibility survey, meaning exercises asked developers to engage in the assigned tasks acting as experienced professionals who were employed in health care work and familiar with the challenges of relying on electronic health records (EHR) data.

**Fig. 2:**
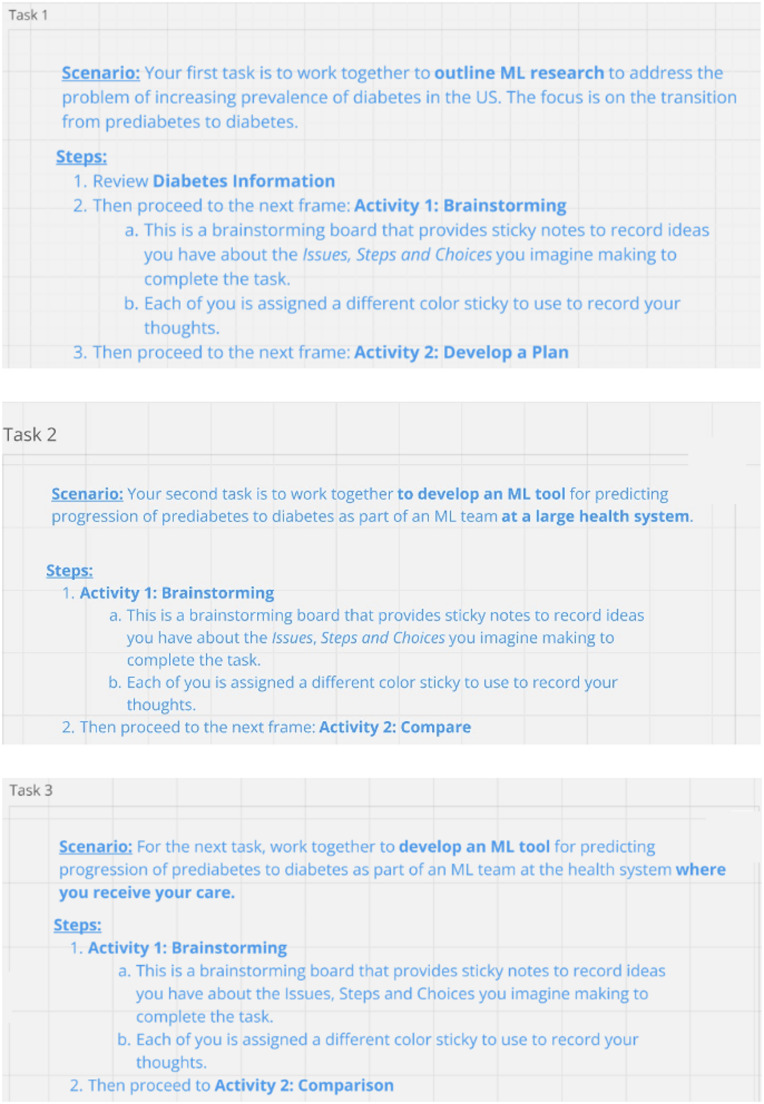
Three task descriptions on Miro board

The GEs utilized Miro^®^, a virtual collaborative whiteboard platform, to facilitate discussion. The Miro board outlined three main tasks for the participants to complete as a group. The first task (T1) asked participants to design an ML-based research project concerning the transition from prediabetes to diabetes in the U.S. and the second (T2) asked participants to develop an ML tool for a health system to address the same problem. The third task, T3, was designed as an intervention. T3 duplicated T2 with one exception: the scenario for T3 added the condition that the hypothetical health system that had employed developers to design T2’s tool was also the system from which they, as employees, received their own health care. This addition was meant to highlight to MLDs the potential for their design decisions’ to have impacts that extend beyond the immediate, intended consequences of an ML feature or choice. We structured T3 in this way to observe whether and how an *imagine-self* framing in the context of ML work elicited responses predicted by the literature, that is: would an *imagine-self* perspective—through the fiction of MLDs themselves also being patients—encourage MLDs to speculate more about the consequences for patients, and others, of the hypothetical tools they were designing (see Fig. 2).

### Participant selection

We identified MLDs who used machine learning to create tools for precision medicine. The rationale for this sample was to focus on a set of practitioners who used similar methods and data sources that included EHRs. We first identified organizations developing precision medicine AI/ML health care tools by searching large, established academic and industry databases, including LexisNexis, ISI, Crunchbase, and ACM with search terms such as “machine learning,” “precision medicine,” “personalized medicine,” and “healthcare.” These searches generated lists of researchers and their organizational affiliations. Using these affiliations, we searched LinkedIn to identify data scientists for study participation by filtering searches for relevant positions, such as data scientist, software engineer, or manager. To increase the diversity of our recruitment pool we also searched professional organizations’ websites, such as those for Black in AI, Women in Machine Learning and Data Science, Black Women in AI, and Diversity and AI. To minimize bias while choosing individuals to contact for possible participation, we contacted individuals based on the order in which they appeared in an organization’s results. Online Resource 1 provides a full list of these organizations as well as additional detail on recruitment procedures.

Drawing on these lists, we sent out a screening survey to establish respondents’ study eligibility with questions about their U.S.-residency status, length of work history in health care-related ML, current employment in health care ML, and, whether in that employment, they utilized EHR data. See Online Resource 2 for the screening survey. Failure to meet this final criterion was the most common reason respondents did not meet eligibility criteria. Based on survey responses, we invited eligible potential participants to participate in the study. If they agreed to participate, we obtained written consent. Of the 47 who took the eligibility survey, 28 were asked to join the study. Of these, 22 agreed and 20 completed the study. Participants were invited periodically until four participants were confirmed for a particular date and we convened five such groups. Study participation included a 90-minute GE session via Zoom^®^, as well as pre- and post-GE interviews with the individual participants, each lasting 30–45 min. Data reported here is drawn from the GE transcripts and from screening survey results. Analysis of the interview data will be reported separately.

### Data collection

Using Miro, GEs opened with an exercise designed to familiarize participants with each other and to review Miro navigation instructions that we had previously distributed. Participants were then directed to the appropriate Miro board location and introduced to the first activity for T1. Activity one directed MLDs to think individually about what considerations would be required to complete the task and, using Miro’s virtual sticky note function, to record these ideas. To facilitate subsequent use of the sticky note comments (SNCs) we advised participants to limit each note to one idea. After several minutes of Activity one, we directed participants to Activity two on the Miro board. Activity two asked MLDs to collectively discuss their SNCs, to expand, revise or delete content and finally to order the notes into an approximate sequence they might follow to complete the task—that is, to develop a plan. T2 and T3 followed a similar order. At two points over the course of the GE, MLDs were asked to compare their responses across tasks. GE sessions were conducted by PLS and MKC, with TEdB online for technical support and other team members observing. All GEs were conducted virtually with both audio and video recorded. Recordings were transcribed verbatim and reviewed for accuracy and to remove identifying information.

### Ethics approval

Stanford University’s Institutional Review Board (IRB) approved this study (#50813). Upon completion of all study phases, including the GE and two interviews, MLDs received an electronic $500 gift card for their participation.

### Data analysis

GE sessions generated two types of data: SNCs and audio recordings. Both types of data were minimally processed and cleaned. Audio recordings were downloaded from Zoom and transcribed. Transcriptions were reviewed and analysis was facilitated using NVivo^®^ qualitative data analysis software. SNCs were imported into NVivo for qualitative coding and into Excel for quantitative analysis.

#### Data analysis—goals and objectives

Analysis of each data set served distinct but complementary purposes toward fulfilling the goal of expanding understanding of how ethical considerations might relate to MLD comments or decisions. Ascertaining the place of ethical considerations in MLD comments and choices requires knowing something about the broader context within which such considerations might emerge. Thus, to provide context for analysis of ethics-related exchanges, SNC analysis was directed toward identifying the full range of topics and questions MLDs introduced as they worked on assigned tasks. The premise that awareness of consequences for others of one’s actions is foundational to ethical conduct directed thematic transcript analysis to exchanges that alluded to an outcome or effect associated with actions or choices mentioned by the MLDs; in other words, how did MLDs express awareness that their actions or choices might have consequences for others. Thematic analysis was directed toward examining to what groups consequences might occur, and, relative to MLDs, what kind of status or agency was ascribed to the interest holder.

#### Data analysis—procedures

**Fig. 3:**
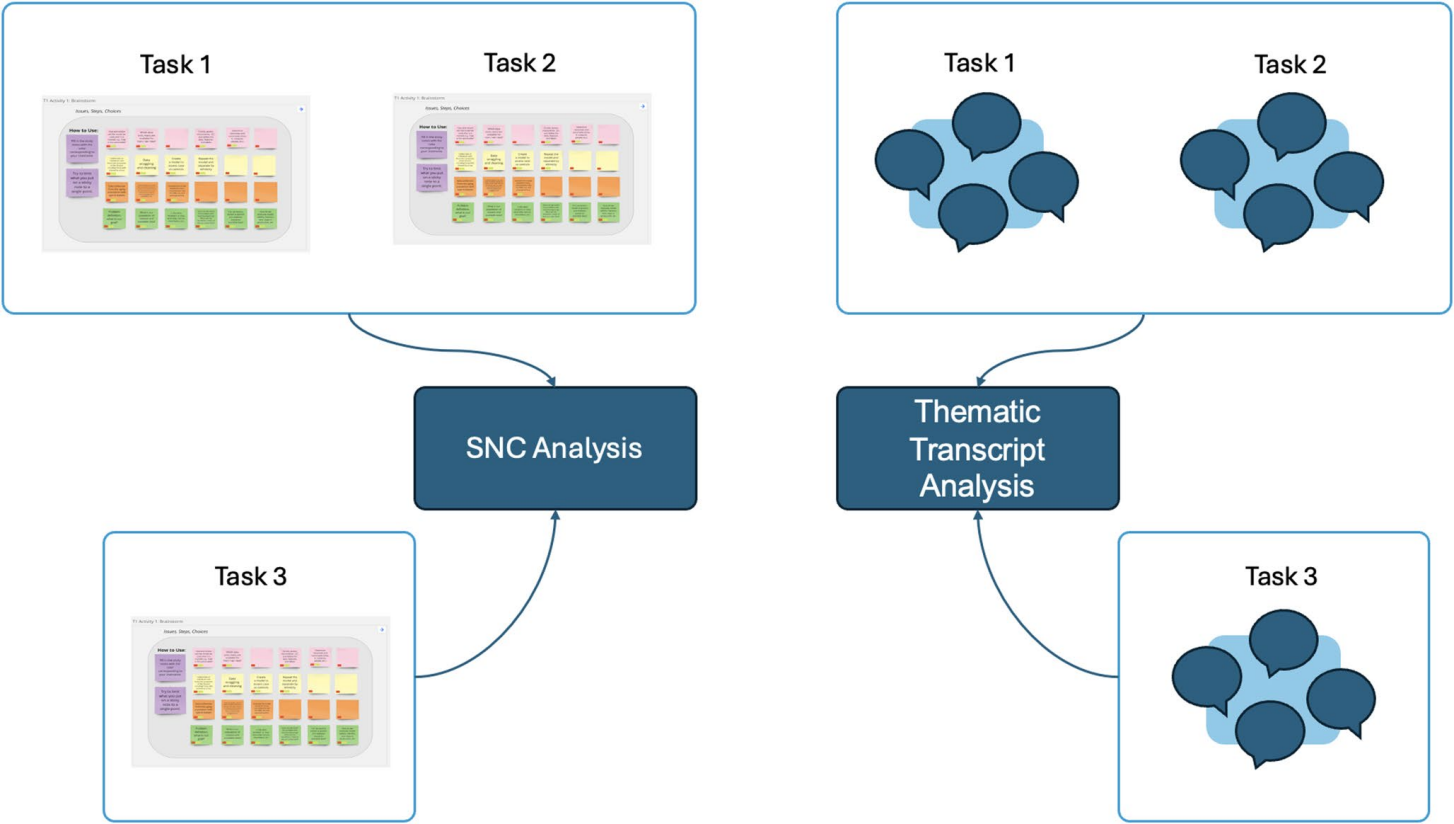
Data sources for analysis

Qualitative analysis procedures for the two datasets were similar and followed standard methods. Analysis of both datasets began with open-ended reading, memoing, and discussion by all study team members. This work generated sets of provisional codes. Coding of SNCs sought to identify primary topics and questions present in MLD exchanges. Thematic transcript analysis focused on identifying themes and patterns pertinent to MLD awareness of potential consequences of ML research and tools, which reflected the ethics-related focus of our research. Working in pairs, team members applied the provisional codes to each data set (see Fig. 3 of data sources for analysis). The team as a whole then reviewed, revised, and finalized the pair-generated coding. Both SNCs and transcript passages could be assigned multiple codes. Finalized codes were entered into NVivo. Quotes are reproduced below with corrected typos and minor adjustments for clarity. SNCs are reproduced as written by the MLDs, with minor corrections indicated by brackets.

SNCs were exported from Miro and formatted as a spreadsheet dataset. All SNCs were aggregated across the five groups for quantitative analysis to identify overall patterns in design considerations across the full sample, rather than compare frequencies between groups. Codes were operationalized as non-mutually exclusive indicator variables (e.g., coded = 1, not coded = 0). Individual codes were grouped together based on similar characteristics to form five non-mutually exclusive indicator variables representing the following finalized aggregate categories: (1) Problem Framing; (2) Data Work; (3) Stakeholders; (4) Implementation; and (5) Social and Ethical Issues. We imported coded SNCs into statistical analysis software (Stata/SE version 15.1) to conduct descriptive and bivariate analyses. Chi-square and Fisher’s exact tests were utilized to identify the difference between the proportion of coded comments across GE tasks (e.g., T1 versus T2, T1 versus T2 versus T3). To characterize the magnitude of these associations, we calculated Cramer’s V values for each chi-square test.

## Results

Based on previous work [31, 32], we hypothesized that participants, accustomed through education and employment to collaborate in teams for work tasks, would accept our fictional work-similar framing and, relying on implicit knowledge, would endeavor to fulfill the assigned tasks in a manner roughly equivalent to conduct produced under actual work conditions. MLD conduct—willingness to follow directions about time, topics, and procedures, as well as comments about the experience—suggest participants accepted the GE framework. Presentation of research results begins with T1 and T2 results. T3 results will be presented separately, following T1 and T2 SNC results. This order allows us to relate findings in a narrative form roughly parallel to participants’ experience of the tasks as they unfolded. Most of the passages quoted here are identified with ID numbers that were assigned to individuals as they enrolled in the study, starting with 1001 and extending to 1021. SNCs generated during the second part of T1 and T2 were not recorded individually but by one GE member acting as scribe for the group. These comments are identified with abbreviations GE1, GE2 and so on, up to GE5, corresponding to each of the five GE cohorts.

### Participant demographics

A total of 20 people who worked on U.S.-based health care ML-related projects involving EHR data were recruited for participation in the study. Participants represented a range of professional settings, including private companies, government agencies, and academic institutions. The typical participant was White (45%), male (75%), worked in either an academic setting (40%) or private company (35%), and had 3–10 years of experience working in AI (75%). In terms of gender representation, a quarter of participants were women (*n* = 5; 25%), aligning with broader trends in AI [47]. For additional demographic information, see Online Resource 3.

### SNC analysis T1 & T2

**Fig. 4:**
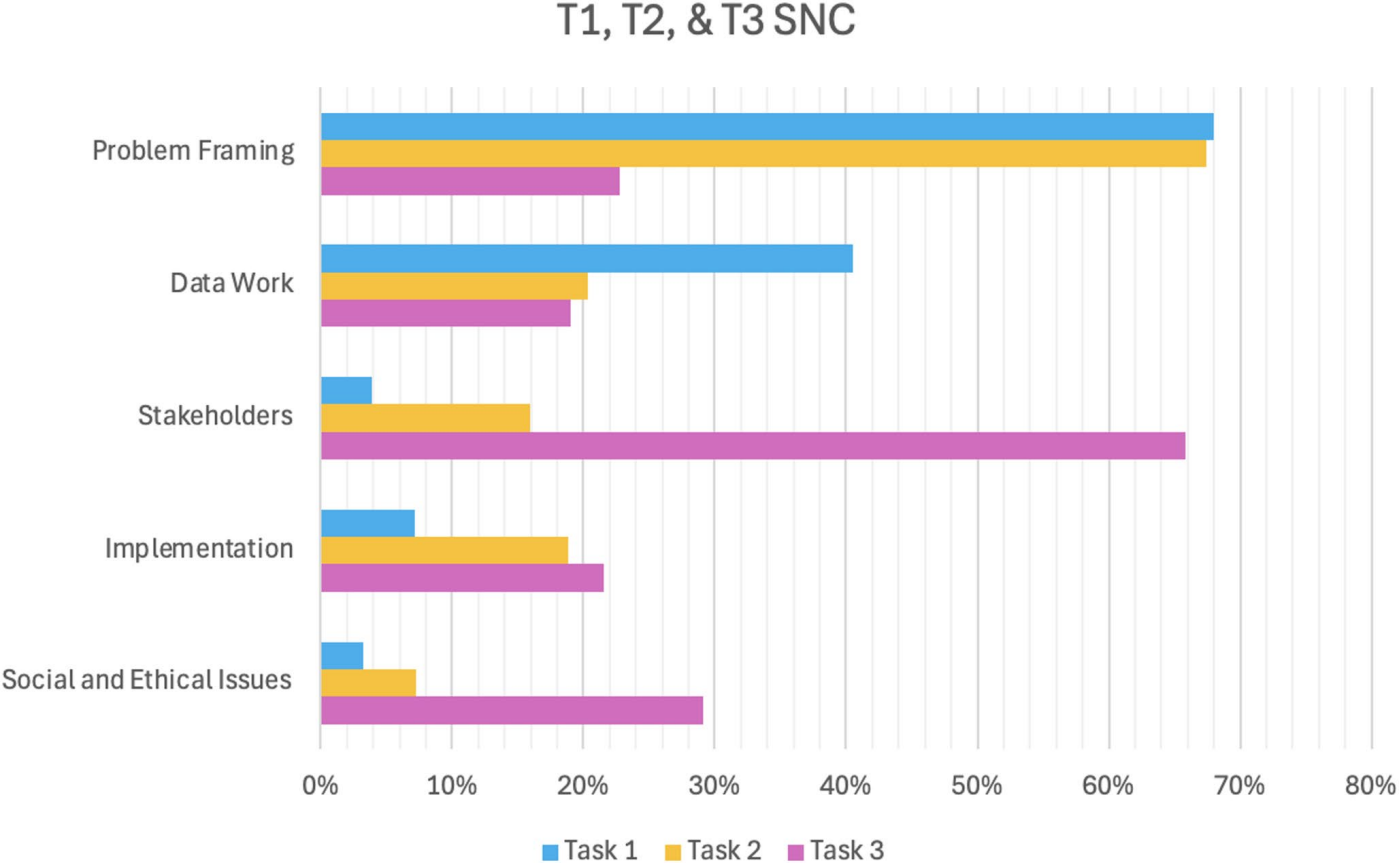
Distribution of SNCs coded to five categories (Problem Framing, Data Work, Implementation, Stakeholders, and Social and Ethical Issues) by task. The bar graph shows percentage of SNCs in each category (*N* = 370)

Quantitative coding of SNCs provided a descriptive overview of design considerations across tasks, serving as context for the subsequent thematic analysis of transcripts. Over the course of completing T1 and T2, MLDs produced a total of 370 codable SNCs related to topics and concerns MLDs discussed. Constrained by the sticky note format and our advice to restrict SNCs to one idea per sticky note, SNCs were similarly brief across tasks, ranging typically from one to six words. Comments often took the form of directives—suggesting a need to consult, gather, identify, or compare. Few comments advised against taking the action or decision named, rather framing it neutrally as the next needed step. Coding distributed these comments into five categories: Problem Framing, Data Work, Implementation, Stakeholders, and Social and Ethical Issues. Figure 4 illustrates the relative proportions of SNC comments that fell under each category per task. While Problem Framing and Data Work made up the majority of comments in T1 and T2, Stakeholders made up the largest proportion of T3. Social and Ethical Issues comments made up less than 10% of comments in T1 and T2 compared with almost 30% for T3.

Table 1 provides descriptions of these categories and examples from T1 and T2. Statements relating to Problem Framing appeared more often than those for the remaining four categories, making up more than half the SNCs for both T1 and T2, while comments relating to Social and Ethical Issues were least common in both tasks. Online Resource 4 provides more detailed accounts of these categories. Here we present descriptions of how MLDs handled each task in its own terms.

**Table 1:** SNC coding category descriptions with T1 and T2 examples

Category	Description	Examples
Problem Framing	Problem Framing involves actions or procedures mentioned as important to building ML. Encompasses comments about the project’s scope, such as desired features, problem definition, and information gathering	Clearly define the hypotheses. Biologically how does this transition look like? Statistically, how should the transition be estimated? [1003, T1] Clarify the goal with the health system – to reduce incidence, to reduce cost, or to improve treatment outcome. [1019, T2]
Data Work	Data Work includes phases and types of activities mentioned as necessary to fulfill ML data needs, specifically referencing, collecting, preparing, and using data to build and train models	What is our population of interest and [available] data? [1017, T1] Identify relevant stakeholders, understand incentives, and develop political capital to unblock data access and deployment [1016, T2]
Implementation	Implementation encompasses actions to initiate, launch, and put into operation an ML tool or research project. Programmatic or bureaucratic, these actions are external to actual ML development	Define intentions for completed work (research v internal use. If research, which journal, etc.) [GE5, T1] Meet infrastructure support team to understand and plan integration challenges [1008, T2]
Stakeholders	Stakeholder comments express interest in concerns or sentiments of individuals or groups participating in the ML process other than MLDs	work with PCP team to see what are the missing pieces (education / food security …) [1012, T1] What is the experience level of the users of the tool [1020, T2]
Social and Ethical Issues	Social and Ethical Issues encompasses comments about ML’s intersection with society or that were attributed to the algorithms themselves	Is the data reliable? i.e. bias, [diversity], nature, cleanliness, etc. [1017, T1] Iterate and improve w.r.t. interpretability, speed, performance, fairness, data representation privacy, etc. [1001, T2]

#### SNC analysis T1

T1 asked MLDs to design ML-directed research to address the health care challenge posed by the high rate of conversion in the U.S. population from pre-diabetes to diabetes, a disease that can lead to serious health problems. As they worked on T1, MLD comments focused primarily on framing the problem they were to work on and on clarifying their goals in relation to the stipulated research context. One MLD stated, “So I guess first step is we need to choose a project. There’s many potential projects” [1001]. To refine their goal, MLDs floated various ideas such as defining the tasks’ scope “as a public health/implementation science/informatics problem” [GE5], or asking if the “ultimate goal” should be: identify “risk factors, design implementation plans, or communication strategy, or identify at-risk populations?” [1019]. Several MLDs noted that choosing among research goals required gathering information to build a better understanding of the health care problem at issue. One asked “What are the baselines / state of the art in the field?” [1017], and others proposed conducting a literature review [1008]. MLDs’ comments also took into consideration the eventual use of the research results, as in: “Define intentions for completed work (research v internal use. If research, which journal, etc.)” [GE5].

Methods and design questions contributed to Problem Framing, as in: “How can we tackle the problem with machine learning?” [1017]. Such comments often prompted responses concerning Data Work, the second most common T1 SNC topic. These included basic questions: “How to define T2D [Type 2 Diabetes]?” [1007], as well as appeals for certain types of data including, “pre-dispositional” and “actionable data“ [GE1] and “educational attainment” [1011]. Concerns about availability of data and MLD access to it figured prominently in these exchanges: “What type of data is available?” [1007]; “What dataset works best for needs?” [1010]; and “Where is the data coming from?” [1009]. Of note, it was in comments about data access and needs that two important topics received the scant attention they were given. Data quality was mentioned as a feature to consider when choosing a dataset: “Is the data reliable? i.e. bias, diversity, nature, cleanliness, etc” [1017], and the IRB was mentioned as a factor that might determine which datasets are accessible: “What type of datasets available, IRB issues” [GE2].

#### SNC analysis T2

T2 asked MLDs to create an ML tool that could address the pre-diabetes to diabetes conversion problem, as in T1. T2 generated fewer comments than T1, possibly because having just completed T1—similarly anchored in diabetes and organized through Miro whiteboards—some issues were taken for granted by the group. Issues MLDs considered while working on T2 were generally similar to those reviewed in T1, as was importance accorded to each, based on frequency. The emphasis accorded each topic in T2 comments and interests expressed therein, however, differed.

In T2 discussions, as in T1, Problem Framing comments were most common. Among those seeking to clarify and refine T2’s goals, there were two types. The first concerned the project’s goal formally, as in “Define the testing hypothesis and collect the data based on the hypotheses” [1018]. The second type differed in that they brought tool users into deliberations, as in “Clarify the goal with the health system - to reduce incidence, to reduce cost, or to improve treatment outcome” [1019] and “meet with stake holders to understand context in which the tool is used (ie point of care, passive alert, pop health)” [1008]. Comments directed at choosing, assessing, and iterating possible models drew considerable attention, some of which echoed interest in the tool’s users by voicing concern for the final output of the tool and potential interventions: “Determine how model results are actionable, e.g., interventions” [1016].

Data-related comments dealt with data availability and ways to collect data, often highlighting anticipated obstacles to data access: “Identify relevant stakeholders, understand incentives, and develop political capital to unblock data access and deployment” [1016]. Attention to preprocessing steps, in particular data labelling, validation, and cleaning was evident. Some expressed desire to evaluate the tool in a clinical context: “Consider the clinical setting to evaluate on validate the data” [1015], indicating awareness of potential weaknesses in chosen datasets but rarely framed these concerns explicitly as data quality issues. Illustrating MLDs’ T2 awareness that the point of a tool was its use, comments about this task took into account staging and preparing for its deployment: “Operationally, what are the different substeps and milestones/deadlines/checkpoints,” [1003] as well as later-stage roll out requirements as in: “Try to launch model in a limited setting and then try to iterate and improve” [GE2].

#### Thematic transcript analysis T1 & T2

Thematic transcript analysis was directed toward identifying material related to MLDs’ awareness of consequences for others of choices related to GE assigned tasks and to ML generally. Emic examples (meaning those the speaker would recognize as pertaining to ethics) and etic (referring to statements identified as ethics-related through analysis) were coded. Passages identified as referring to a consequence included an identifiable choice or action and an interest holder on which the action had a consequence. These passages were reviewed to identify two features: (1) the interest holders named as objects or recipients of possible consequences; and, (2) relative to MLDs, what kind of relationship was ascribed to the interest holder.

##### 4.2.3.1 Awareness of consequences

Exchanges between MLDs suggested an awareness of the potential consequences of their actions and decisions in relation to four different interest holders: patients, colleagues, users, and sponsors. Patients were described as sources of EHR data, members of health care systems, members of various patient communities such as healthy or ill patients, or social groups such as Hispanic patients. This interest holder category also included statements where MLDs referred to themselves as a patient. Colleagues referred to project-related stakeholders named as necessary to facilitating a project’s completion, such as research collaborators, clinician domain experts, and health care information technology personnel. Users as an interest holder encapsulated potential users of the project tool within a health system, typically referring to either clinicians or patients. Sponsors included regulators or health systems, as interest holders that involve managing proposals for health care machine learning tool use, whether in relation to regulatory or payment adoption.

MLDs positioned themselves relative to interest holders in various ways, which we examine here in terms of levels of engagement and responsibility. Analysis identified four ways MLDs positioned themselves toward interest holders: presumed, collaborative, deferential, and reflexive. *Presumed* refers to relationships where MLDs discussed interest holders’ supposed goals or preferences without indicating any direct engagement with those interest holders. For example, this includes statements about extracting patient information from EHRs or other datasets that omit mention of consulting patients about this action. *Collaborative* relationship statements expressed interest in seeking input or assistance from colleagues or experts, beyond an MLDs’ immediate team. *Deferential* refers to statements that expressed MLDs inclination to accede to preferences or interests of stakeholders whom MLDs assumed had more importance or authority than the MLDs. *Reflexive* showed MLDs’ adoption of an *imagine-self* perspective—recognizing themselves as potential interest holders and considering how their tools could impact their own lived experiences. Table 2 provides examples of how MLDs discussed the consequences of their design choices for different interest holders and the nature of their relationships with them. The majority of T1 statements regarding consequences to specific interest holders concerned patients as potential sources of EHR data. T2 statements had a similar focus but evidenced higher frequency of statements about collaborative relationships with colleagues and of statements expressing deference to users and sponsors. In certain cases, MLDs displayed deference by ensuring relevant stakeholders such as regulators were consulted prior to key decision making, aligning with regulatory requirements. Notably, the majority of MLDs who discussed consequences for patients in T1 and T2 did so through a presumed relationship, positioning patients primarily as data sources. Statements demonstrating reflexive engagement—considering how they as interest holders themselves might be affected—were rare, with only one across T1 and T2. In the absence of an *imagine-self* framing, MLDs did not spontaneously engage in PT or position themselves as interest holders. A more detailed breakdown of interest holder references and relationship types across tasks appears later in the analysis.

**Table 2:** Examples of consequence passages analysis: interest holders and relationship

Example passages	Interest holders	Relationship
“Yeah. I mean, related to the data. I think there is another, there are two other things, two other potential sources. One is the real world data from things like the insurance claims and population studies because we talk a lot about the risk factors in terms of lifestyle and the other thing, I mean history and else. Another is actually you know variables or other self-reported data because that tracks the lifestyle. So I just want to mention in those cases the good thing is usually you can get a very large number of sample size like we can have studies with like 16 million people. But then at the same time, usually the data, quality of data, you know data completeness is usually bad. So we need to take those into account when dealing with that during those kinds of studies. So I think this is still going back to the question based on what they want to achieve. But I mean if lifestyle is one thing we want to look into, that’s congregated, then maybe we have to collect” [1019]	Patient	Presumed
“So for me, I would say those metrics are important. Are we talking about… I’m not the doctor here, so I don’t know what… if I went into a doctor’s appointment and they assessed how diabetic I am… of course that’s a metric, we want to know that. We also have those secondary metrics, like weight, exercise, age, all very good, and then I’ll jump to the conclusion. I think I would like… one of our goals might be to give someone a… like a report card that says how likely are you to develop… to go from prediabetic to diabetic would be one thing, you know, just a simple prediction based on assessment of where you are today, and another could be what’s the likelihood of… you know, four things you could do. What’s the likelihood of any of those making a difference on outcome?” [1005]	Patient	Reflexive
“So initial[ly], basically, this would be their needs, so [what] do they need, and then the second part when we talk to them we would want to show them what we’ve done to increase their buy in, so they kind of get excited, they want to use it. And then we would have to train them and make sure they know how things work, and what is the expectation” [1012]	User	Deferential
“I think too that probably all of us have had to use tools made by somebody else, or we have had to share our code or models with other people and it’s always such a pain if you don’t know how to use their tool or they don’t know how to use your tool. So you always want to just prevent that as much as possible where there’s, try to make sure that there’s no incompatibilities so that, or the worst thing is that they just don’t use your tool at all because it doesn’t actually work for what they need. So all those considerations should be made ahead of time” [1020]	Colleague User	Collaborative Deferential
“It’s largely as the same, if I’m designing for a large health system, my interest in pop health is maybe more acute, but I still have a lot of the same issues around data collection and datasets that are available, mostly it stays the same. My only thought is, in a large hospital system you’re going to be much more constrained by budget than time” [1011]	Sponsor	Deferential

### Intervention

We then conducted an intervention with T3, where we employed an *imagine-self* frame in the context of ML work to see if MLDs would take on the perspective of themselves as patients and consider the consequences of the hypothetical tools they were designing for patients. First, we will describe the results of the SNC analysis of T3 to provide the broader context of considerations MLDs were thinking through in their design decisions. Second, we will summarize the quantitative findings of our chi-square analysis of the distribution of SNC categories across all three tasks, highlighting significant differences. Lastly, a comparison of our thematic analyses across all three tasks will be presented.

#### SNC analysis T3

When MLDs were asked to take on the perspective of patients, their comments primarily centered on stakeholders, with a secondary emphasis on social and ethical considerations. Common themes across Stakeholder comments included the necessity for design that aligns with patient preferences, involvement of stakeholders in the development process, and considerations of cost and accessibility for patients or end-users. Among the various stakeholders considered, patients were the most frequently mentioned. Social and Ethical Issues were often mentioned through the lens of the patient experience. For example, a recurring topic was bias with comments such as “I will give [additional] focus to fairness and populations that are not similar to me.” [1017]. MLDs frequently mentioned the accuracy of the model and asked questions about outcomes or risks involved, such as “automatic diagnosis takes a risk to trust. What is the backup plan?” [1006]. See Table 3 for further T3 examples of each SNC category.

Compared to T1 and T2, Problem Framing was less common in T3 discussions. When present, Problem Framing discussions largely revolved around modeling. Similar to T2, MLDs considered benchmarking against existing models and validating models in clinical settings. Some comments reflected how adopting a patient’s perspective could shape model design, as in, “If you know you differ in a biologically meaningful way from the majority class, you may be incentivized to make the model generalize better” [1016]. Conversely, some MLDs expressed concerns that their patient perspective could introduce bias into the modeling process: “If I were [a] patient, this will be a major conflict of interest for me --- what I consider important could bias the model as I will be affected by it” [1017]. Regarding data access, MLDs acknowledged their domain knowledge and connections as potential advantages: “Advantages of working in your own health system: maybe you know people who can help you get access to data, collaborate, answer questions, etc” [1020], and “Will work with motivation to resolve this problem and contact the patients to help us during data collection” [1015]. Finally, T3 brought out patient considerations in determining the features of the tool, such as providing the patient with a “triage” [1006] and “patient education material when the progression is fast” [1012].

**Table 3:** SNC coding category descriptions with T3 examples

Category	Description	Examples
Problem Framing	Actions or procedures mentioned as important to building ML, e.g., comments about the project’s scope, such as desired features, problem definition, and information gathering	The ML model should provide me with a triage [1006, T3] Can my experience inform the data collection, testing and model development process? [1017, T3]
Data Work	Phases and types of activities mentioned as necessary to fulfill ML data needs, specifically referencing collecting, preparing, and using data to build and train models	Advantages of working in your own health system: maybe you know people who can help you get access to data, collaborate, answer questions, etc. [1020, T3] Will work with motivation to resolve this problem and contact the patients to help us during data collection [1015, T3]
Implementation	Actions to initiate, launch, and put into operation an ML tool or research project. Programmatic or bureaucratic, these actions are external to actual ML development	Experience of being a patient here could provide useful insight with regard to interventions from the model [1016, T3] Need to pay good attention to HIPAA compliance and de-identification of data [1020, T3]
Stakeholders	Comments expressing interest in concerns or sentiments of individuals or groups participating in the ML process other than MLDs	What does my doctor think of this tool [1011, T3] Ensure the endeavor does not increase wait times, turnaround for lab tests, etc. and otherwise impact patient experience and satisfaction [1016, T3]
Social and Ethical Issues	Comments about ML’s intersection with society or that were attributed to the algorithms themselves	If you know you differ in a biologically meaningful way from the majority class, you may be incentivized to make the model generalize better. [1016, T3] Automatic diagnosis takes a risk to trust. What is the backup plan? [1006, T3]

#### SNC chi-square analysis

As shown in Table 4, Problem Framing was the most frequent category overall, accounting for more than two-thirds of T1 and T2 comments. In contrast, only 22.8% of T3 comments addressed this issue. Data Work was the second most frequent category in T1 and T2, with its frequency changing minimally from T2 (20%) to T3 (19%). Stakeholders, the third most common category in T1 and T2 discussions was, in contrast, T3’s most numerous. Implementation-related comments increased across the three tasks, with the largest gain being between T1 and T2. Social and Ethical Issues were minimal in T1 and T2 but constituted 29% in T3. Statistical analyses (chi-square and Fisher’s exact tests) showed significant differences in proportions of SNCs coded as Problem Framing (*p* < .001), Data Work (*p* < .001), Stakeholders (*p* < .001), Implementation (*p* = .009), and Social and Ethical Issues (*p* < .001) across the three tasks. Effect sizes ranged from small-to-moderate for Problem Framing (V = 0.22) and Data Work (V = 0.25), to moderate for Implementation (V = 0.31), and very large for Stakeholders (V = 0.77) and Social and Ethical Issues (V = 0.69), indicating substantial shifts in how SNC patterns across tasks.

**Table 4:** T1 versus T2 versus T3 SNC coding: chi-square analyses compared category frequency across tasks (Alpha = 0.05)

Category	Total*	Task 1 (*n* = 153)		Task 2 (*n* = 138)		Task 3 (*n* = 79)		χ^2^	Cramer’s V	*p*
		#	%	#	%	#	%			
Problem Framing	217	104	68.0	93	67.4	18	22.8	21.57	0.22	< 0.001
Data Work	105	62	40.5	28	20.3	15	19.0	13.59	0.25	< 0.001
Stakeholders	78	6	3.9	22	15.9	52	65.8	95.61	0.77	< 0.001
Implementation	49	11	7.2	26	18.8	17	21.5	10.05	0.31	0.009
Social and Ethical Issues	38	5	3.3	10	7.2	23	29.1	35.85	0.69	< 0.001

*Some SNCs were assigned to multiple categories; therefore, totals exceed the total number of SNCs (*N* = 370)

#### T3 integrative thematic transcript analysis

**Fig. 5:**
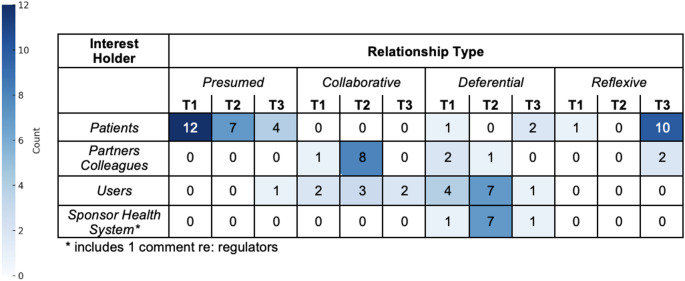
Consequence passage analysis showing number of MLDs referencing interest holders and relationship types across tasks. The color scale represents counts of MLDs from zero (white) to 12 (dark blue)

Figure 5 presents the combined interest holder and relationship type data for T1, T2, and T3, with the numbers indicating the count of MLDs (out of 20 total) who made comments referring to specific interest holders and relationship types. Because some MLDs referred to certain interest holders multiple times, the data is presented by the number of MLDs rather than the total number of comments to avoid overrepresentation. This table provides a comparative view of how MLDs consider the consequences of ML work based on how they treated or prioritized interest holders’ interests across the three tasks. It is not meant to imply causal certainty, but rather to provide illustration of the potential impact of *imagine-self* framing in the context of designing a precision medicine ML tool.

Across the three tasks, there were shifts in both the interest holders mentioned and the nature of relationships with these groups. In T1, the majority of MLDs (12 out of 20) referred to patients in a presumed relationship, characterizing them as sources of information. References to partners/colleagues, users, and the sponsor/health system were less frequent and generally deferential, suggesting MLDs assumed that these interest holders could dictate priorities or decisions. In T2, the number of remarks about interest holders increased, implying that completion of T2 required MLDs to expand their frames of reference. The number of MLDs mentioning partners and colleagues in a collaborative capacity increased (from 1 of 20 to 8 of 20), and included comments about shared decision-making and consulting domain expertise. Mentions of users and the sponsor/health system increased but mostly included deferential comments made by 7 of the 20 MLDs in each category. T2 references to patients changed little from T1 and were categorized primarily as presumed. In both T1 and T2, reflexive comments were rare, with only one participant explicitly situating themselves in a patient’s role.

The relationship type relative to patients changed notably across the tasks. In T1 and T2 comments, MLDs overwhelmingly discussed patients in a presumed relationship, expressing a reflexive stance only once in the T1 remark: “if I went into a doctor’s appointment and they assessed how diabetic I am… of course that’s a metric, we want to know that” [1005]. In contrast in T3, MLDs most frequently made comments adopting a reflexive relationship to interest holders, in particular, to patients. Ten out of 20 participants imagined themselves as patients personally impacted by the tool. The introduction of a PT prompt substantially changed MLDs comments about patients. As one participant mused, “what if I’m the patient, or what if I’m being like directly affected by this model in a way?” [1017]. Rather than relating to patients predominantly as sources of data as in T1 and T2, T3 prompted MLDs to consider the implications of being data subjects. One participant expressed concern about data use and trust: “I wish there was so many guardrails on our data that the data could just help me and not manipulate me” [1005]. In addition, MLDs reflected on their own roles as developers for the first time in T3 (captured in Partners Colleagues - Reflexive). One participant noted their tendency as a developer to prioritize technical aspects over broader considerations, stating, “we tend to focus a lot on, okay, these are the most steps, these are the methods, right. But we don’t like focus enough on framing the question, right, like who are the patients, but also who’s gonna be the user of the model, what’s going to be the, like the data available, the goals, the baselines. So I think sometimes like we as, as practitioners tend to get excited about the methodological aspects in like, and we don’t consider other aspects, right, that are broader sometimes” [1017]. These forms of reflexivity marked a shift from earlier tasks, where MLDs primarily discussed consequences in relation to external groups and rarely acknowledged their own involvement in the design and impact of ML tools on patients.

### Comments indicating impact of GE framework

MLDs made a number of remarks indicating the impact of the GE framework. Several also expressed awareness of how the tasks influenced their thinking, especially when asked to reflect on their experience and whether their design considerations shifted across the three tasks. One MLD stated, “we were putting on different stakeholder hats in each one, so in the first one we were maybe more of the PIs, in the second one more of the engineers and research-… direct researchers” [1001]. Several MLDs indicated that the tasks prompted them to adopt different perspectives. Another participant reinforced this point succinctly: “we changed our point of view” [1003].

Many MLDs described how T3, in particular, prompted them to adopt a patient perspective: “The thing that really changed was I really, instead of being about the science, and dataset, and stuff like that, I really tried to think what would it feel like for a patient to go through whatever we design” [1006]. This perspective increased their motivation to work on the problem and develop tools that were more convenient and useful for patients. Some participants recognized that a strong focus on data could obscure the human element: “I tend to forget about it because I start looking at the data and at some point after, I don’t know, a couple of months working a problem, I kinda forget about some human aspects of it” [1017]. Additionally, one MLD reconsidered their dual role as both a health system employee and a patient: “When I was working in the [large research center], I never considered myself as a cancer patient. But because I was directly working with the physician there and the data comes from patients, I always wanted to go with him for visiting and, you know, contact with the patients. […] The only difference here is that I never considered myself as a patient, but I was involved with their patients for my research” [1021].

Finally, participants expressed how the experience might influence their approach to future work. One MLD highlighted the value of interdisciplinary collaboration, stating, “I haven’t really been doing a lot of, you know, have people with different backgrounds like business and different and then let’s sit in the same room doing practices and exercises like this. I think this is actually very good exercise. And so that we can more be on the same page. I need to borrow some of that later” [1019]. Beyond collaboration, one MLD described how the experience prompted them to rethink aspects of their own work in balancing being data-driven with a broader perspective: “We got really focused on the data more and forgot about exactly what was the big picture really, and I think that’s, personally I have that, and I think something I need to change in my work” [1018]. Overall, MLDs engaged with multiple perspectives throughout the tasks and described how the process led them to reflect on and reassess aspects of their own work.

## Discussion

Ethical conduct is the product of complex, highly contextualized reasoning. Our findings contribute to ongoing discussions about the persistent gap between ethical principles and their practical application in AI ethics. While principles play a critical role by distilling a field’s priorities and values to inform practice over a broad range of actors and settings, translating them into practice remains a challenge. This is the case even for fields such as medicine with long-standing, robust ethical traditions, a fact that AI observers of medical ethics’ apparent success often overlook [14, 48]. This raises the question of whether AI developers—who typically lack ethical training that includes identification of others’ interests or ethical frameworks that are based on fiduciary duties—require alternative strategies to support ethical reasoning in their work.

Our study explored PT as one such strategy, investigating whether prompting MLDs to adopt an *imagine-self* viewpoint could enhance their identification with people who are affected by their design choices. Our findings suggest that machine learning developers working on precision medicine health care models can effectively participate in hypothetical health care machine learning design work that encourages their awareness of potential consequences of health care AI to others. Shilton [49] demonstrated that when computer science students tested new systems on themselves, it led them to consider the ways in which their data could reveal personal information and behaviors. Like our hypothetical design exercises, this internal prototyping creates the potential for personal reflection, which in turn increases sensitivity to values such as privacy and equity and “encourage[s] consensus around those values as design criteria”—what Shilton called values levers [49].

Our GEs relied on developers engaging with scenarios designed to introduce ethical concerns through differing perspectives and discussing their actions or choices. MLDs were willing to follow directions about procedures, topics, and time throughout the exercises and to collaborate with their group, verbalizing their reasoning on their processes and discussing their thoughts with other MLD participants. Tasks asked developers to focus not on choosing specific methods but on articulating development considerations, and then to outline processes by which they would achieve development goals. Although the scenarios were hypothetical, this kind of engagement with others in artificially-constructed GEs captured how MLDs might work in their real-world practice. Prior work by Heger et al. concluded that to bridge a principles-to-practice gap, organizations should develop activities and policies that align with ethical activity across multiple layers of an organization, including within teams [50]. Focusing on AI development teams is essential to ensure that interventions effectively increase MLDs’ awareness of potential harms and cultivate a sense of responsibility for mitigating them.

In the analysis of 370 SNCs, themes emerged about the range of process concerns MLDs considered in model design, and the frequency of different types of comments. Comments most frequently focused on Problem Framing and Data Work considerations in Tasks 1 and 2. When MLDs were instructed to consider themselves patients within the health system in the third task, comments shifted towards Social and Ethical Issues and Stakeholders. Statistical analysis found categorical differences across tasks to be significant. Analysis of the SNC comments provides an understanding of the context with which MLDs make design decisions, creating a foundation for interpreting the thematic transcript data, which enabled more nuanced expression of awareness and consideration of the effects of their model work decisions on others.

Together, the SNC and thematic analyses suggest that as design considerations broadened across tasks, MLDs’ discussions of interest holders and relationships shifted accordingly. Thematic analysis findings revealed developers’ awareness and perspective of different interest holders and how they related to these groups through presumed, collaborative, deferential, or reflexive orientations. Relationship statements expressed MLDs’ assumptions about how, when, and for whom they took into account the needs, rights, or input of others in AI design. Across the tasks, MLDs’ awareness and perspectives on interest holders and relationships changed. In T2, MLDs referenced a broader range of interest holders in comparison to T1, such as health care administration, providers, users, and health care information technology departments, in a collaborative and/or deferential manner. The approach of bridging principles to practice through inclusion of an *imagine-self* frame in the last task, where MLDs were asked to imagine themselves as the patients in the health care system where implementation of the ML tool would take place, then allowed for more in-depth insight into potential consequences through the shifts in their relationships with these interest holders. These findings suggest that the *imagine-self* frame not only made it more likely that MLDs took others’ perspectives and understood the consequences on others, but also that adopting this frame made it more likely that MLDs would see a need to incorporate those others’ perspectives into their design.

Several MLDs were aware of their own transition of perspective. In T1 and T2, MLDs discussed patients primarily in terms of the data their health care produced and treated the relationship as presumed. However, the *imagine-self* frame introduced in the last task generated different kinds of comments: MLDs discussed patients with “I” statements, as the relationship shifted to a reflexive one. This shift in language and relationship demonstrates that the GE framework facilitated PT. MLDs’ awareness of this impact provides additional evidence that the GE approach can lead MLDs to consider the effects of their work on others. Furthermore, MLDs were able to reflect on how participating in the group exercise changed their thinking about ML design and specific ways it might change their work practices in the future.

Previous research found that AI developers distance themselves from moral responsibility for the potential harms associated with application of their models in health care [51]. As a building block to foster a sense of responsibility and ethical conduct, developers must first develop an awareness that their actions’ have consequences for others. Cultivating awareness of different perspectives is most effective when it elicits a participant’s own goals and values. Developing one’s own interest in the outcomes of their decision-making encourages long-term behavior change [52].

Our results do not compare *imagine-self* with *imagine-other* approaches directly. However, they are consistent with previous findings that *imagine-self* instructions are associated with responses deemed socially positive, potentially due to identification with the hypothetical target or recipient [40, 53]. Myers et al. [40] found that in comparison with *imagine-other* and objective instructions, *imagine-self* instructions may be more effective at increasing helping behaviors (actions intended to benefit others). In their study, participants listened to a broadcast and were asked to imagine how they would feel if they were experiencing what had happened to the interviewee. In addition to imagining how they were feeling, this approach also strengthened a psychological sense of connection and shared identity with the interviewee, which in turn further motivated helping behavior. Jang [53] examined charitable giving and found that when participants first took the perspective of a single victim, they felt a stronger psychological connection to multiple victims in similar situations, ultimately leading to increased donations. Research investigating awareness of ethical issues associated with AI and ML among computer science and information science students echoes these results [29]. Further study comparing *imagine-self* with *imagine-other* interventions will be necessary to determine which approach is more effective.

This study has implications for regulatory practices regarding AI in health care. While recent global initiatives, such as the EU AI Act, UNESCO’s Recommendation on the Ethics of AI, and IEEE 7000 reflect growing international attention to responsible AI, this study focuses on the experiences of U.S.-based developers working within a largely self-regulatory environment. Accordingly, our findings should be interpreted within the U.S. context. Prior regulation has focused on company or organizational culture as a scaffolding for best practices within AI development for health care tools and devices. The Food and Drug Administration had initially taken a novel regulatory approach to ML-based software as a medical device through a precertification pilot program, where emphasis was placed on a company’s process and “culture of quality and organizational excellence” [54]. Since then, further documents have been released, including guiding principles and recommendations for a predetermined change control plan for AI-enabled software device functions [55–58]. On January 6, 2025, draft guidance was released for lifecycle considerations pertaining to submissions for AI-enabled medical devices [59]. However, the state of AI regulation continues to evolve, and there is a need to rely more on self-regulation of MLDs in developing a state of awareness of the moral considerations of their software design choices and their agency in mitigating such harms within their teams at companies and organizations they are working for.

Our study had several limitations. One main limitation of the research is that there were 20 study participants of MLDs in five groups. Because the participants were U.S.-based, these results may not generalize to developers working in other contexts where regulatory environments may differ. However, the MLDs who participated in the study had significant experience in ML and included diverse perspectives, given our recruitment efforts at professional organizations such as Women in Machine Learning and Data Science. Since the tasks were completed in a fixed order, increasing familiarity with the scenario may have contributed to some of the observed shifts. Another limitation of the study is the potential for social desirability bias. We designed the group exercises purposefully to take into consideration approaches to minimize social desirability bias, such as choosing not to ask direct questions about ethics and to limit mention of ethics in the group exercises script. In addition, our study lacked long-term follow up beyond exit interviews with each participant. Furthermore, we used hypothetical scenarios not related to participants’ actual work, with groups of people who had not worked together before, so we do not know whether scenarios based on actual projects or groups of collaborators would yield similar results. Our future research intends to test the effectiveness of the group exercise in actual work situations. Lastly, because SNCs were aggregated across all five participant groups during quantitative analysis, our approach may have obscured variation within and/or between groups. However, as the aim of our analysis was to characterize the overall range of considerations, any changes in participants’ patterns were described at the individual level.

## Conclusions

As AI continues to evolve, regulatory frameworks have lagged, leading organizations to rely on internal ethical oversight, potentially leaving MLDs with the responsibility to identify and mitigate potential harms but without a clear mandate or guidance. While many scholars have proposed ethics codes or guidelines for developers, the literature has largely overlooked the realities of developers’ day-to-day routines, attitudes, and decision-making. Our study contributes to bridging the gap from principles to practice by demonstrating that developers can effectively engage in ethical reflection through structured group design exercises. When prompted through an *imagine-self* framing, asking developers to view themselves as potential patients impacted by their own tools, participants identified with the patient perspective and demonstrated increased awareness of interest holders affected by their design decisions. Importantly, this awareness could motivate MLDs to reconsider design decisions from the patient perspective. Several MLDs made comments acknowledging this shift in perspective, providing further evidence that PT may foster ethical reflection that informs their future work. By creating space for collaborative, reflective engagement, such exercises show promise not only as a research method but also as an instructional tool. This approach could inform future educational materials, training programs, and ethical development practices aimed at cultivating responsible AI design from within project teams. Future research is needed to assess whether and how developers integrate these reflections into real-world practices over time. In particular, implementing similar exercises within multidisciplinary teams could help evaluate how ethical awareness is shaped by team interactions and organizational culture and to what extent the insights gained in structured settings translate to actual actions.

## Supplementary Information

Below is the link to the electronic supplementary material.

Supplementary Material 1

Supplementary Material 2

Supplementary Material 3

Supplementary Material 4

## Data Availability

Miro board data and group exercise transcripts contain identifiable personal data and are not publicly available. De-identified excerpts relevant to the findings are provided within the article and supplementary material. Additional information may be available from the corresponding author upon reasonable request.
