# Supplementary material for: Machine learning for precision medicine: promoting value considerations through perspective-taking hypothetical group design exercises
Source: AI Ethics. 2026 Feb 1;6(1):127. doi: 10.1007/s43681-025-00973-5 (PMC12862023; doi:10.1007/s43681-025-00973-5)
Supplement: Supplementary file 1 — Supplementary Material 1 [file 43681_2025_973_MOESM1_ESM.pdf]

Machine learning for precision medicine: Promoting value considerations through hypothetical group design exercises

AI and Ethics

Corresponding author contact details will be provided after acceptance.

Online Resource 1 Study recruitment additional details

LexisNexis	Search criteria: machine learning OR artificial intelligence OR AI and healthcare and precision medicine OR personalized medicine OR individualized medicine; News; English; United States; Results must include: omic. Results: 51 articles, 26 Companies mentioned. 4 of the companies returned professionals' contact info in Nexis Company Dossier.
Crunchbase	Search criteria: Keywords: precision medicine, individualized medicine, personalized medicine; Industry: artificial intelligence, intelligent systems, machine learning, natural language processing, predictive analytics. Results: 61 companies. Of those, 25 have company pages on Linkedin, with 261 data scientists as employees.
Women in Machine Learning & Data Science	Search criteria: women involved in a health-related company or project based on their Linkedin profiles that were provided on the website and Linkedin company profile. Results: 2 contacts.
Black in AI	Search criteria: United States; Keywords: medicine, health, healthcare. Manually sorted through matches from the website member list and looked up potential contacts on Linkedin. Had to look up some individuals on their institution's website or personal professional website. Results: 21 contacts from 20 distinct institutions, which were a mix of academic and industry.
ACM	Search criteria: Keywords: personalized medicine, individualized medicine, precision medicine, genomics; Group: Official Association for Computing Machinery; Location: United States. Results: 188 total results, with 18 data scientists with Linkedin profiles.
ISI	Search criteria: (machine learning OR artificial intelligence OR AI) and healthcare and (precision medicine OR personalized medicine OR individualized medicine); English; USA. Results: 387 articles. Pulled out emails for all authors, resulting in 567 authors. Searched on LinkedIn for profiles of the authors using their names and organizations.
Other	Search criteria: precision medicine OR personalized medicine OR individualized medicine in other groups: Black Women in AI, Diversity and AI, R-Ladies, Women in Artificial Intelligence, Blacks in Technology, Black in Data. Results: 5 contacts.
