# Supplementary material for: Machine learning for precision medicine: promoting value considerations through perspective-taking hypothetical group design exercises
Source: AI Ethics. 2026 Feb 1;6(1):127. doi: 10.1007/s43681-025-00973-5 (PMC12862023; doi:10.1007/s43681-025-00973-5)
Supplement: Supplementary file 2 — Supplementary Material 2 [file 43681_2025_973_MOESM2_ESM.pdf]

**Machine learning for precision medicine: Promoting value considerations through hypothetical group design exercises**

*AI and Ethics*

Corresponding author contact details will be provided after acceptance.

**Online Resource 2** Screening survey for study recruitment

Start of Block:

Q0 Name: ________

Q1 Preferred email (to contact you only in regard to this study) ________

Q2 Preferred phone number (to conduct interviews over the phone) ________

Q3 Are you over 18 years old?
- o Yes (1)
- o No (2)

Q4 Are you currently employed in machine learning related work, or have you been in the past 6 months?
- o Yes (1)
- o No (2)

Q5 Is this machine learning related work conducted in the US?
- o Yes (1)
- o No (2)

Q6 Does your machine learning related work concern healthcare?
- o Yes (1)
- o No (2)

Q7 In your machine learning healthcare related work, do you work on applications related to diagnosing or treating patients that require you to analyze EHR data?
- o Yes (1)
- o No (2)

Q8 What is the highest degree you have received?
- o BA/BS (1)
- o MA/MS (2)
- o MPH (3)
- o MSW (4)
- o PhD (5)
- o MD (6)
- o Other (7) ________

Q9 In what field was this degree?
- o Computer and information systems (e.g., Data Science, Computer Science) (1)
- o Mathematics (e.g., Statistics, Bioinformatics) (2)

- Biological science (e.g., Biology, Genetics) (3)
- Social sciences (e.g., Anthropology, Political Science) (4)
- Physical science (e.g., Physics) (5)
- Engineering (e.g., Bioengineering) (6)
- Business e.g., Healthcare Administration) (7)
- Other (8) ________

Q10 In what year did you receive this degree? ________

Q11 Since completing your degree in Q7, have you been primarily:
- Working for a private company (1)
- Working in an academic setting (2)
- Working for local, state, or federal government (3)
- Working toward an additional degree? If so, please fill in what degree and in which field: (4)

________

Q12 For how long have you worked in AI?
- Less than a year (1)
- Between 1-3 years (2)
- Between 3-5 years (3)
- Between 5-10 years (4)
- Between 10-20 years (5)
- More than 20 years (6)

Q13 For how long have you worked in **healthcare-related** machine learning
- Less than a year (1)
- A year or more (2)

Q14 How do you describe yourself?
- Male (1)
- Female (2)
- Trans Male/Trans Man (3)
- Trans Female/Trans Woman (4)
- Genderqueer/Gender Non-Conforming (5)
- Different Identity (6)
- Prefer not to answer (7)

Q15 Are you of Hispanic/Latino/Spanish origin?
- Yes (1)
- No (2)

Q16 How would you describe yourself? (Select all that apply)
- ▢ American Indian or Alaska Native (1)
- ▢ Asian, South Asian, or Southeast Asian (2)
- ▢ Black or African American (3)
- ▢ Native Hawaiian or Other Pacific Islander (4)
- ▢ White (5)
- ▢ Middle Eastern or North African (6)
- ▢ None of the above describe me (8)
- ▢ Prefer not to answer (7)

End of Block
