# Supplementary material for: Machine learning for precision medicine: promoting value considerations through perspective-taking hypothetical group design exercises
Source: AI Ethics. 2026 Feb 1;6(1):127. doi: 10.1007/s43681-025-00973-5 (PMC12862023; doi:10.1007/s43681-025-00973-5)
Supplement: Supplementary file 3 — Supplementary Material 3 [file 43681_2025_973_MOESM3_ESM.pdf]

Machine learning for precision medicine: Promoting value considerations through hypothetical group design exercises

AI and Ethics

Corresponding author contact details will be provided after acceptance.

Online Resource 3 Participant demographic information additional details

Participant Characteristics	(n=20)	%
**Race/Ethnicity***		
Asian, South Asian, or Southeast Asian	4	20%
Black or African American	3	15%
Middle Eastern or North African	3	15%
White	9	45%
Hispanic/Latino/Spanish origin	1	5%
None of the above describe me	1	5%
Not answered/Prefer not to answer	3	15%
**Gender**		
Female	5	25%
Male	15	75%
**Work Setting**		
Working for a private company	7	35%
Working for local, state, or federal government	2	10%
Working in an academic setting	8	40%
Working towards an additional degree	3	15%
**Length of Work in AI**		
Between 1-3 years	3	15%
Between 3-5 years	8	40%
Between 5-10 years	7	35%
Between 10-20 years	1	5%
More than 20 years	1	5%

*Participants selected all that applied
