# Supplementary material for: Machine learning for precision medicine: promoting value considerations through perspective-taking hypothetical group design exercises
Source: AI Ethics. 2026 Feb 1;6(1):127. doi: 10.1007/s43681-025-00973-5 (PMC12862023; doi:10.1007/s43681-025-00973-5)
Supplement: Supplementary file 4 — Supplementary Material 4 [file 43681_2025_973_MOESM4_ESM.pdf]

**Machine learning for precision medicine: Promoting value considerations through hypothetical group design exercises**

*AI and Ethics*

Corresponding author contact details will be provided after acceptance.

**Online Resource 4** SNC categories descriptions and examples

| Category | Description and examples |
|---|---|
| Problem Framing | Problem Framing involves actions or procedures proposed as important to building ML, encompassing comments about the scope of the project, such as desired features, problem definition, and information gathering. Participants were concerned with defining the hypothesis and the goals or purpose of the tool. This included identifying the target population and needs of end users. Comments were frequently phrased as questions, such as “What is our population of interest and available data?” [1017]. They considered different avenues for study design, such as clinical trials, observational studies, and self-reported data. Participants were also concerned with how to estimate the transition between disease states: “Contextually, how do physicians distinguish prediabetes and diabetes” [1020]. In addition, participants considered the final output of the model and potential interventions, for example, “offer patient/provider actionable course,” and “patient education material when the progression is fast” [1012]. Existing tools or frameworks and different modeling approaches were brought up, taking into account model complexity and the constraints of the customer: “What ML/statistical methods do they support/not support? (e.g. some people don't like Neural Networks)” [1020]. They considered various performance metrics and measures by which to evaluate the model such as feasibility, generalizability, and sensitivity versus specificity. Finally, participants focused heavily on model validation, comparing their results with other models, and how to iterate and improve the model, such as “feed back results to start of process to refine data, model” [GE4]. |
| Data Work | Data Work includes phases and types of activities mentioned as necessary to fulfill ML data needs, specifically referencing collecting, preparing, and using data to build and train models. Many comments identified types of data/metrics that would be most useful or relevant for the project. Examples of data types were “blood glucose” [1004], “predispositional data” [GE1], “actionable data” [GE1], and “educational attainment” [1011]. Participants were frequently concerned with how to access this data: “Identify relevant stakeholders, understand incentives, and develop political capital to unblock data access and deployment” [1016]. They explored various avenues to collect data, for example “pull research data from OMOP data model to represent clinical data” [1008]. In addition, participants mentioned a wide variety of preprocessing steps, in particular data labelling, validation, and cleaning. Participants also mentioned data quality in these conversations, however comments were made infrequently. Finally, participants were concerned with how to evaluate it in a clinical context: “Consider the clinical setting to evaluate on validate the data” [1015]. |
| Implementation | Implementation encompasses actions to initiate, launch, and put into operation an ML tool or research project. Programmatic or bureaucratic, these actions are external to actual ML development. Comments related to implementation covered the full span of a project but clustered at early and late stages. Early-stage comments named steps needed to get a program off the ground, asking for example, “i” [1009], or noting the need to “assemble [a] team” [GE1] or to determine the “sponsor” [1010] and “cost/who is paying” [1012]. Steps to move the product forward generated comments about potential stakeholders. For the ML research project this might mean audience for the resulting research, as one participant explained, there is a need to “Define intentions for completed work (research v internal use. If research, which journal, etc)” [GE5]. For the tool development projects, stakeholder comments concerned intended adopters or users, usually personnel in healthcare settings. These comments often overlapped with comments about other steps needed to roll out a project. One note described a next step as “Try to launch model in a limited setting and then try to iterate and improve” [GE2]. Finally, the implementation category also includes statements made about regulations possibly relevant to the fictional ML work. Few comments addressed this topic. Those that did mentioned it in the context of steps required to access data, such as asking: “Any IRB or other regulation of data access? How long it will take to complete this?” [1014]. |
| Stakeholders | Stakeholder engagement in ML projects is crucial for ensuring that the developed tools meet the diverse needs and expectations of all parties involved, such as patients, clinicians, and healthcare administrators. We captured such comments by categorizing comments that referenced stakeholder demands and expectations attributed to these stakeholders, and settings and user experience (UX) requirements crucial for the successful development and |

| | |
|---|---|
| | implementation of ML systems. Common themes across comments coded as AP included the necessity for UX design that aligns with user preferences, involvement of stakeholders in the development process to ensure contextual appropriateness, and considerations of cost and accessibility for end-users. Our analysis also revealed a strong emphasis on direct interactions with various stakeholders to gather insights and refine the ML tool. For instance, references to UX highlight the importance of making tools intuitive and accessible, reflecting a broader need for user-friendly technologies in healthcare settings. Participants expressed concerns about the cost implications for patients and the need for tools to be explainable, as seen in comments like, "The ML model should be explainable to me... I should understand how the ML models came with the singularities to categorize me in class 1 or 2" [1006]. Furthermore, repeated references to meetings with stakeholders such as IT engineers, administration, and healthcare providers underscore the collaborative nature required in ML development. The notion of frequent and focused interaction contrasts with other development aspects, suggesting that stakeholder engagement is not only foundational but requires ongoing attention to adapt and respond effectively to the evolving needs and contexts of healthcare delivery. |
| Social and Ethical Issues | We grouped codes about social or ethical issues associated with ML's intersection with society or that were attributed to the algorithms themselves in the Social and Ethical Issues category, which was the least populated of the five categories. Included here are issues that the AI literature typically labels ethical, as captured by the following SNC that suggested that the group should consider: "interpretability, speed, performance, fairness, data, representation, privacy, etc" [1001]. Privacy was a commonly mentioned issue, in comments supporting attention to "privacy and security" [1006] by, for example, having a "secure login" [1005] for the proposed diabetes tool. Also included were the handful of comments participants offered concerning the risks or benefits to patients of the proposed ML work. One comment, for example, suggested talking to a "group of patients to see what can be beneficial for them" [1012], and another asked if "automatic diagnosis" offered by ML posed a risk to patient trust and asking, if so, "What is the backup plan?" [1006]. Finally, comments related to bias and fairness were the most common type in this category, such as "If you know you differ in a biologically meaningful way from the majority class, you may be incentivized to make the model generalize better." [1016] |
